# Gastroprotective Effect of Hexanic Extract of *Heliotropium indicum* Against Ethanol-Induced Gastric Lesions in a CD1 Mouse Model

**DOI:** 10.3390/plants13233449

**Published:** 2024-12-09

**Authors:** María Elena Sánchez-Mendoza, Yaraset López-Lorenzo, Ximena del Rocío Torres-Morales, Leticia Cruz-Antonio, Daniel Arrieta-Baez, Jazmín García-Machorro, Jesús Arrieta

**Affiliations:** 1 Escuela Superior de Medicina, Instituto Politécnico Nacional, Plan de San Luis y Díaz Mirón, Colonia Casco de Santo Tomás, Miguel Hidalgo, Mexico City 11340, Mexico; mesmendoza@hotmail.com (M.E.S.-M.); yarlop_2310@outlook.com (Y.L.-L.); torres.morales.ximena.rocio@gmail.com (X.d.R.T.-M.); jazzgama81@gmail.com (J.G.-M.); 2 Facultad de Estudios Superiores Zaragoza, Universidad Nacional Autónoma de México, Av. Guelatao No. 66, Colonia Ejército de Oriente, Iztapalapa, Mexico City 09230, Mexico; letycruza@gmail.com; 3 Centro de Nanociencias y Micro y Nanotecnologías, Instituto Politécnico Nacional, Unidad Profesional Adolfo López Mateos, Av. Luis Enrique Erro s/n, Mexico City 07738, Mexico; darrieta@ipn.mx

**Keywords:** *Heliotropium indicum*, gastroprotection, antioxidant activity, antisecretory activity

## Abstract

Peptic ulcers result from an imbalance between protective factors (e.g., prostaglandins, nitric oxide, and sulfhydryl groups) and aggressive risk factors (e.g., consumption of non-steroidal anti-inflammatory drugs, alcohol, or tobacco) regarding the gastric mucosa. While various existing treatments aim to relieve pain, repair the ulcer, and prevent its recurrence, they often produce undesirable side effects. The *Heliotropium indicum* (*H. indicum*) plant has been utilized as a traditional medicine due to its gastroprotective activity. In this study, we identified the compounds responsible for the gastroprotective activity of the hexanic extract of *H. indicum* in an ethanol-induced damage model, in addition to determination of the activities of prostaglandins, nitric oxide, and non-protein sulfhydryl groups, along with the antisecretory and antioxidant activities (i.e., concentration of malondialdehyde and activities of the enzymes superoxide dismutase, catalase, and glutathione peroxidase). We found at least two groups of compounds that are responsible for this activity, namely 1-acyl-glycerol components and retinyl β-glucuronide derivatives. In conclusion, a mixture of compounds responsible for the gastroprotective activity of *H. indicum* was isolated from its hexanic extract, and non-protein sulfhydryl groups were implicated in its mechanism of action.

## 1. Introduction

Peptic ulcers are lesions of the digestive tract that often occur in areas in contact with gastric juice and can reach the *muscularis mucosae*. In some cases, they can cause perforation and subsequent leakage of intestinal contents, leading to peritonitis [1]. Depending on where the lesion is located, ulcers can be classified as esophageal, gastric, or duodenal ulcers. This condition affects 10% of the global population at some point in their lives. Peptic ulcers are the result of an imbalance between the protective factors (e.g., the mucus/bicarbonate layer, prostaglandins, and nitric oxide) and risk factors (e.g., gastric juice; consumption of non-steroidal anti-inflammatory drugs (NSAIDs), alcohol, or tobacco) for the mucosa gastric [2]. According to Sharifi-Rad et al., although peptic ulcers rarely lead to death, their symptoms can cause discomfort, disrupt daily activities, and consequently cause mental distress in patients [3].

The present treatments for gastric ulcers are aimed at alleviating pain, repairing the ulcer, and preventing the recurrence of peptic ulcer disease. Nonetheless, their mechanisms of action differ, as certain drugs inhibit gastric acid secretion by inhibiting the H^+^/K^+^ ATPase, blocking H_2_ receptors, or stimulating the secretion of mucus and bicarbonate to safeguard the mucosa [4]. The use of H^+^/K^+^ ATPase inhibitors, such as omeprazole, holds great promise in the treatment of gastric ulcers; however, their prolonged use can have significant adverse effects, including the inhibition of vitamin B12 absorption, which can lead to irreversible neurological damage, anemia, hypergastrinemia [5], acute myocardial infarction [6], and pancreatic cancer [7].

The above indicates the need to look for new therapeutic alternatives with minimal adverse effects. In this sense, medicinal plants are an important source for obtaining new active substances [8]. *H. indicum* is a plant used in traditional medicine to treat wounds, eye infections, common menstrual problems, nervous disorders, kidney diseases, and fever alleviation. Previous studies have also demonstrated its gastroprotective activity in a gastric lesion model induced by NSAIDs [9], and its antisecretory activity in a pyloric ligation model [10]. However, none of these studies have revealed the compound(s) responsible for these biological activities. Recently, our working group reported that one of the compounds responsible for the gastroprotective activity of *H. indicum* was (*E*)-ethyl-12-cyclohexyl-4,5-dihydroxydodec-2-enoate, which was isolated from the dichloromethane extract. Furthermore, this study demonstrated that the hexanic extract had considerable gastroprotective activity [11]. Therefore, the present study aimed to identify the compound or compounds responsible for the gastroprotective activity of the hexanic extract of *H. indicum*.

## 2. Results

### 2.1. Gastroprotective Effect of Hexanic Extract

**Figure 1:**
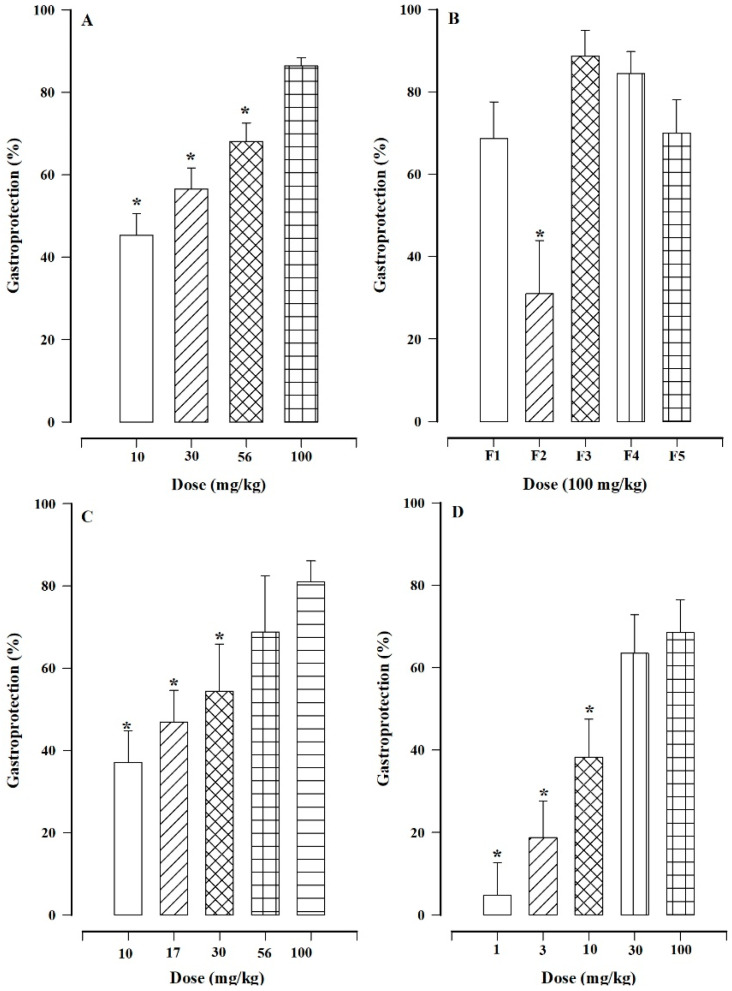
The gastroprotective effects of hexanic extract (**A**), hexanic extract fractions (**B**), mixture of compounds from fraction F3 (**C**), and carbenoxolone (**D**) on gastric lesions caused by ethanol in mice. Bars represent the mean ± SEM (n = 7). * *p* < 0.05 based on the Kruskal−Wallis test followed by Dunn’s multiple comparison or vs. dose 100 mg/kg.

The hexanic extract of *H. indicum* significantly reduced gastric lesions resulting from the administration of ethanol in a dose-dependent manner. From Figure 1A, it is evident that the greatest effect was obtained with a dose of 100 mg/kg (86.45 ± 1.99% gastroprotection), followed by doses of 56, 30, and 10 mg/kg (68.09 ± 4.49, 56.47 ± 5.19, and 45.25 ± 5.27% gastroprotection, respectively). The results obtained at doses of 10, 30, and 56 mg/kg were significantly lower when compared with the results obtained with the 100 mg/kg dose.

### 2.2. Evaluating the Fractions of the Hexanic Extract

With regard to the evaluation of the fractions of the hexanic extract, the outcomes are depicted in Figure 1B. Fractions F1, F3, F4, and F5 provided significant protection against gastric harm resulting from the administration of ethanol, indicating the presence of multiple active compounds in the hexanic extract. However, fractions F3 and F4 performed slightly better than fractions F1 and F5. The results of the mixture of compounds in F3 are presented in Figure 1C, which show that its effect is dose-dependent, reaching 80.99 ± 5.12% gastroprotection as the maximum effect with a dose of 100 mg/kg. Similarly, carbenoxolone exhibited a dose-dependent effect, achieving its maximum effect at a dose of 100 mg/kg (Figure 1D). When comparing the results obtained from carbenoxolone regarding those from the mixture of compounds, it can be seen that the mixture of compounds was more effective than carbenoxolone.

### 2.3. Analysis of the Mixture of Compounds

**Figure 2:**
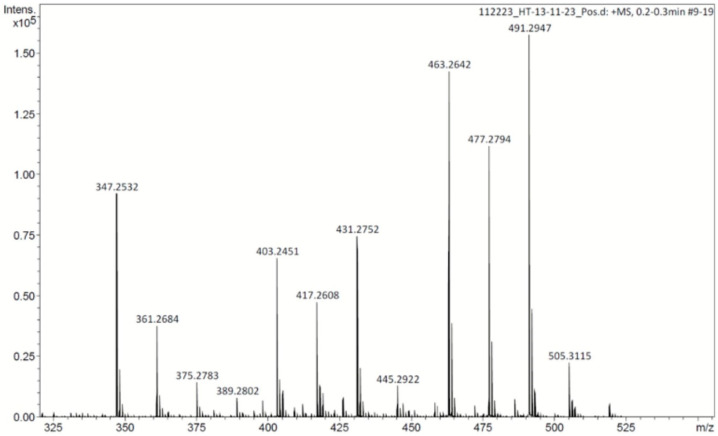
Electrospray ionization mass spectrometry (ESI-MS) of the active fraction (F3).

**Figure 3:**
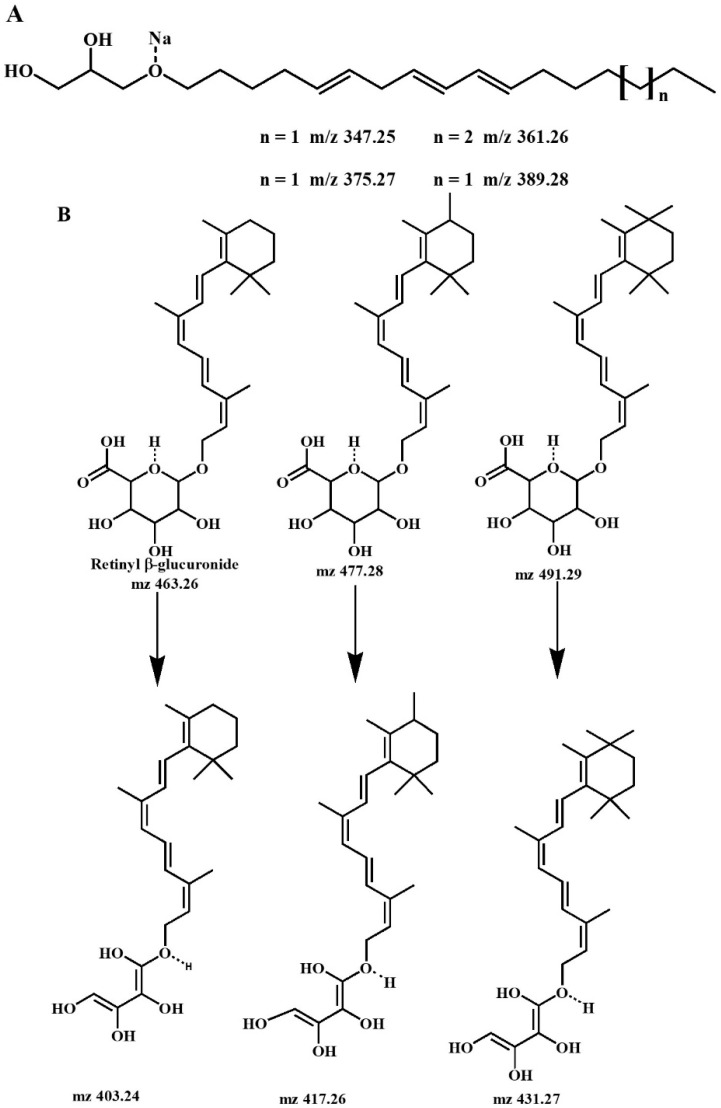
First (**A**) and second (**B**) groups of compounds identified from the active fraction, according to the ESI-MS analysis.

The active fraction (F3) was analyzed using ESI-MS. According to its ESI spectrum, the F3 fraction contained at least two compound groups (Figure 2). For the first group of compounds, ESI (+) showed a molecular ion at *m*/*z* 347.2532 [M+Na]^+^ (Calc *m*/*z* 347.2562), which is consistent with the molecular formula C_20_H_36_O_3_. This compound and those observed at *m*/*z* 361.26, 375.27, and 389.28 were assigned to a 1-acyl-glycerol compound, as shown in Figure 3A. The second group of compounds belonged to retinyl β-glucuronide derivatives (*m*/*z* 463.26, 477.28, and 491.29), as shown in Figure 3B. ESI (+) revealed a molecular ion at *m*/*z* 463.2642 [M+H]^+^ (Calc. *m*/*z* 463.2696), which is consistent with the molecular formula C_26_H_39_O_7_. A set of intermediate compounds were assigned to *m*/*z* 403.24, 417.26, and 431.27, according to Figure 3B. These compounds could be related to a possible decarboxylation in the glucuronide group of the retinyl β-glucuronide. Structures were confirmed using MS/MS electrospray ionization (ESI) mass spectrometry. The fractionation of compounds and their relative percentages in the active fraction are detailed in Table 1.

### 2.4. The Roles of Prostaglandins, Nitric Oxide, and Sulfhydryl Groups in the Mechanism of Action

**Figure 4:**
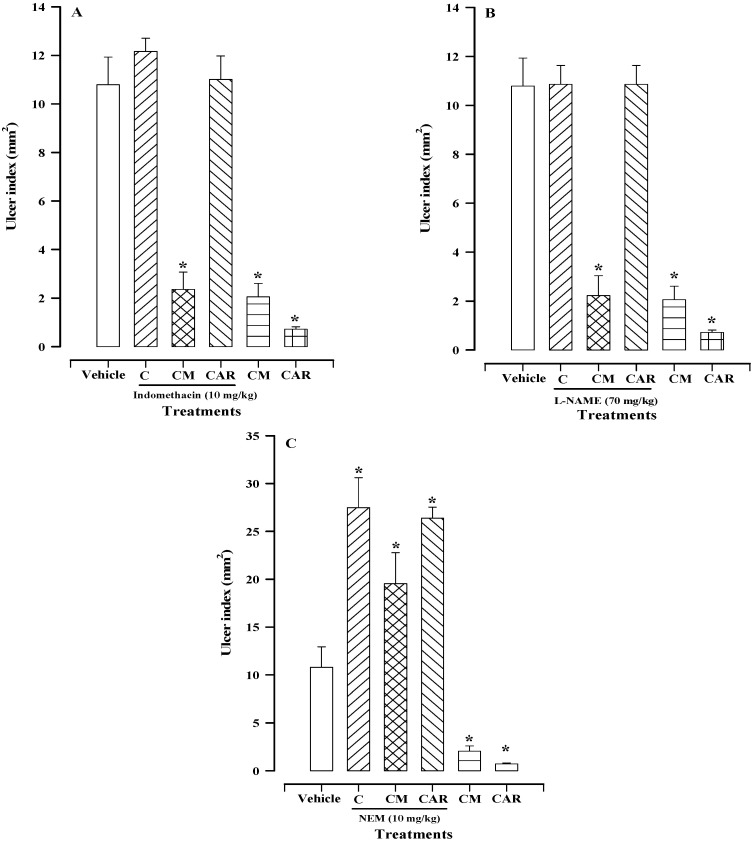
Effects of mixture of compounds (CM) and carbenoxolone (CAR) on gastric lesions induced by ethanol in mice treated with indomethacin (**A**), N^G^-nitro-L-arginine methyl ester (**B**) (L-NAME), or *N*-ethylmaleimide (**C**) (NEM). C denotes control groups for the distinct inhibitors. Bars represent the mean ± SEM (n = 7). * *p* < 0.05 vs. the respective control, based on the Kruskal−Wallis test followed by Dunn’s multiple comparison.

Prostaglandins—specifically PGE_2_—play a fundamental role in the defense of the gastric mucosa. The secretion of mucus and bicarbonate depends on these factors, and an increase in blood flow, among other activities. The administration of indomethacin—a non-specific inhibitor of cyclooxygenases (COX1 and COX2)—therefore inhibits the production of prostaglandins. In Figure 4A, the ulcer index was similar in the groups with vehicle, control, and carbenoxolone (previously treated with indomethacin) (10.79 ± 1.14, 12.16 ± 0.54, and 11.02 ± 0.96 mm^2^, respectively). However, in the groups treated with the mixture of compounds (CM) with or without pre-treatment with indomethacin, the ulcer index was reduced to 2.35 ± 0.71 and 2.05 ± 0.55 mm^2^, respectively, without finding a significant difference, suggesting that prostaglandins may not be involved in its mechanism of action. When compared with the group treated with carbenoxolone plus indomethacin, a significant difference was found with respect to CM.

It is well known that nitric oxide protects the gastric mucosa by increasing blood flow. However, the administration of N^G^-nitro-L-arginine methyl ester (L-NAME), a nonspecific inhibitor of nitric oxide synthases, did not alter the gastric protection of the mixture of compounds, as shown in Figure 4B. Figure 4B shows similar ulcer index values in the group with vehicle, in the control group treated with L-NAME, and the group treated with L-NAME and subsequently carbenoxolone, with values of 10.79 ± 1.14, 10.86 ± 0.76, and 10.86 ± 0.76 mm^2^, respectively. In the group treated with CM alone or previously with L-NAME, the ulcer index did not significantly differ. This suggests that nitric oxide does not participate in its mechanism of action. The ulcer index also decreased with carbenoxolone treatment, without a significant difference, compared with CM.

Non-protein sulfhydryl groups aid in preserving the integrity of the gastric mucosa, particularly when reactive oxygen species are involved in tissue damage. As shown in Figure 4C, the administration of *N*-ethylmaleimide (NEM)—a blocker of non-protein sulfhydryl groups—induced the ulcer index, with values of 27.49 ± 3.15, 19.53 ± 3.24, and 26.38 ± 1.14 mm^2^ in the control, CM, and carbenoxolone groups, respectively. Pre-treatment with NEM resulted in the inhibition of the gastroprotective effect of the mixture of compounds, indicating the involvement of non-protein sulfhydryl groups in its mechanism of action.

Regarding carbenoxolone, the results agree with those previously reported in the literature, as prostaglandins, nitric oxide, and sulfhydryl groups do participate in its mechanism of action [12,13,14].

### 2.5. Effects of Treatment on Malondialdehyde (MDA), Superoxide Dismutase (SOD), Catalase (CAT), and Glutathione Peroxidase (GPx) Activities

**Figure 5:**
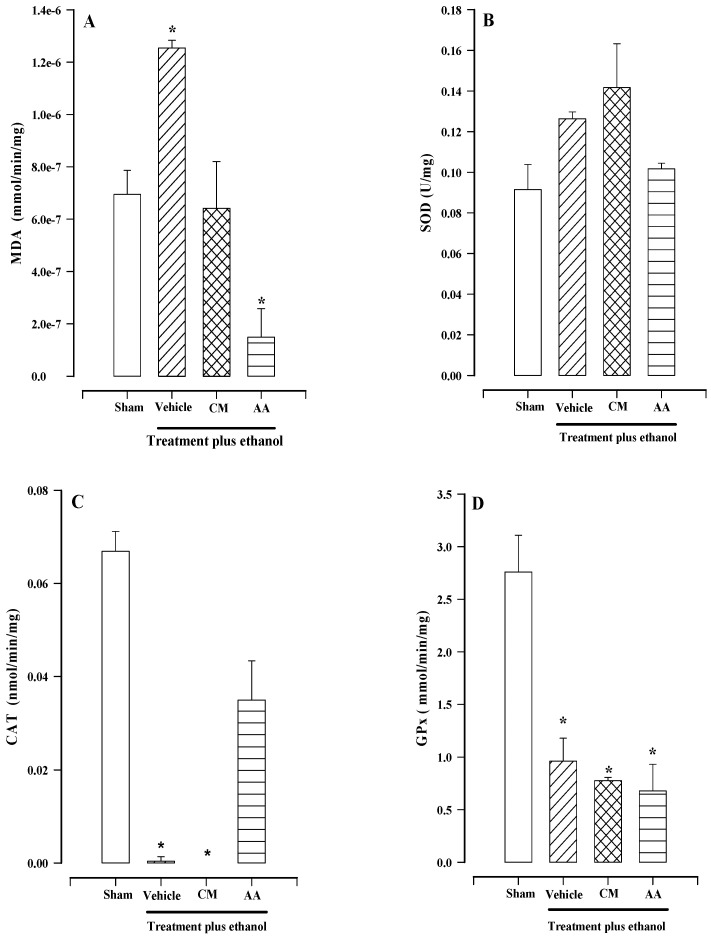
Effect of the mixture of compounds on the activity of MDA (**A**), SOD (**B**), CAT (**C**), and GPx (**D**) on gastric damage caused by ethanol in rats. Mixture of compounds (CM), ascorbic acid (AA). Bars represent the mean ± SEM (n = 7). * *p* < 0.05 vs. the respective sham, based on the Kruskal−Wallis test.

The basal concentration of MDA was 6.94 × 10^−7^ ± 0.918 × 10^−7^ mmol/min/mg in the sham group, which increased when ethanol was administered (1.25 × 10^−6^ ± 0.0296 × 10^−6^ mmol/min/mg). Upon conducting an analysis of the data and comparing the two groups, no significant differences were observed (Figure 5A). The MDA concentration was slightly reduced when the mixture of compounds was administered, as shown in Figure 5A. However, the reduction was not statistically significant compared with the sham group. The administration of ascorbic acid (AA), as an antioxidant, reduced the concentration of MDA to 1.49 × 10^−7^ ± 1.08 × 10^−7^ mmol/min/mg, making it the group with the lowest concentration. In this case, a significant difference was detected.

SOD is a ubiquitous antioxidant enzyme that catalyzes the conversion of the superoxide radical to hydrogen peroxide (H_2_O_2_), providing protection against reactive oxygen species. Figure 5B shows that the concentration of the SOD enzyme increased when CM was administered (0.14 ± 0.021 U/mg) compared with vehicle and AA (0.126 ± 0.003 and 0.10 ± 0.002 U/mg, respectively); however, there was no significant difference when compared with the sham group (0.09 ± 0.012 U/mg).

CAT is an essential antioxidant enzyme, which is present in most aerobic organisms. Catalase is an enzyme that breaks down two molecules of hydrogen peroxide into one molecule of oxygen and two molecules of water. Catalase activity was detected in the sham and AA groups with values of 0.069 ± 0.0042 and 0.035 ± 0.008 mmol/min/mg, respectively (Figure 5C); however, catalase activity was minimal in the vehicle group, and it was not detected in the CM group.

The GPx family of enzymes comprises antioxidant enzymes that serve as essential selenoenzymes in mammals. They belong to the same class of heme-free thiol peroxidases as peroxidases and catalyze the reduction of H_2_O_2_ or organic hydroperoxides to water or corresponding alcohols, thus reducing their toxicity. The GPx enzyme was detected within the sham group at a concentration of 2.75 ± 0.35 mmol/min/mg; however, in the vehicle, CM, and AA groups, it was detected at lower concentrations (0.961 ± 0.218, 0.77 ± 0.031, and 0.678 ± 0.253 mmol/min/mg, respectively) compared with the sham group (Figure 5D). No significant differences were observed between these groups.

### 2.6. Antisecretory Activity

One of the main aggressors of the gastric mucosa is the secretion of hydrochloric acid by parietal cells. The pH of the gastric fluid after administration of the mixture of compounds did not differ significantly from that observed in the vehicle control group. However, the gastric volume in the mixture of compounds group was decreased significantly compared with the control group (Table 2). In contrast, the reference drug omeprazole augmented the pH, and the gastric volume exhibited a similarity to that of the control group, as depicted in Table 2. The above results suggest that antisecretory activity may not be a mechanism of action for the mixture of compounds.

## 3. Discussion

Peptic ulcers are sores in the gastroduodenal mucosa, which can cause severe stomach pain and gastrointestinal bleeding. Peptic ulcer disease refers to conditions that affect the lower esophagus, upper duodenum, and lower stomach [15]. The prevalence of peptic ulcers is approximately 10% of the world population, affecting approximately 4.5 million people annually in the United States [16].

Drugs currently used to treat gastric ulcers have not solved the issue, with all having adverse effects to a greater or lesser extent. This warrants the search for new drugs with similar efficacy and minimal adverse effects; in this regard, medicinal plants may serve as a crucial source of new drugs. *H. indicum* is a medicinal plant for which gastroprotective activity has already been demonstrated, and it has even been possible to isolate (*E*)-ethyl-12-cyclohexyl-4,5-dihydroxydodec-2-enoate as being responsible for this activity from a dichloromethane extract [11]. In the current study, it was demonstrated that the hexanic extract had significant gastroprotective activity, with the administration of the hexanic extract at various dosages revealing a dose-dependent effect (Figure 1A). The results indicate that the extract protects against damage caused by the administration of ethanol.

During the fractionation of the hexanic extract, fractions F1, F3, F4, and F5 exhibited significant gastroprotective activity, indicating that the hexanic extract contains more than one active compound. Fractions F3 and F5 were the most active, as shown in Figure 1B. However, as F3 yielded more, it was subjected to column chromatography, resulting in the isolation of a white powder. Upon evaluation, the gastroprotective effect of this powder displayed a dose-dependent effect on par with that of carbenoxolone.

ESI-MS analysis of the white powder revealed that it was composed of two groups of compounds (Figure 2): the first corresponded to a molecular formula of C_20_H_36_O_3_, which was assigned as components of 1-acyl-glycerol (Figure 3A), while the second group belonged to retinyl β-glucuronide derivatives, which are consistent with the molecular formula C_26_H_39_O_7_ (Figure 3B). A group of intermediate compounds was also detected—compounds that probably derive from the decarboxylation of the glucuronide groups of the retinyl group of β-glucuronide (Figure 3B). According to the relative percentage of the active mixture, the β-glucuronide derivatives had the highest percentage (Table 1) and, so, the activity was most likely due to this type of compound, although this hypothesis should be further corroborated. On the other hand, it is important to note that no gastroprotective activity has previously been demonstrated for any of these compounds. Thus, this would be the first report of such activity for components of 1-acyl-glycerol and retinyl β-glucuronide derivatives.

Prostaglandins—primarily PGE_2_—are produced from arachidonic acid by cyclooxygenases (COX), which are present throughout the gastrointestinal tract. They are known to regulate various functions within the gastrointestinal tract, including acid and bicarbonate secretion, mucus production, and mucosal blood flow. These actions contribute significantly to the safeguarding of the gastric mucosa [17,18]. Indomethacin—a non-specific COX inhibitor—was not able to reverse the gastroprotective effect of the mixture of compounds (Figure 4A). Based on the observed results, prostaglandins do not appear to be involved in the mechanism of action of the compound mixture. Meanwhile, regarding carbenoxolone, its effect was reversed (Figure 4A), suggesting that prostaglandins are a part of its mechanism of action. This result is in accordance with findings previously reported in the literature [19].

Nitric oxide (NO) plays an important role in maintaining the integrity of the gastric epithelium and mucosal barrier. It modulates the gastric ulcer healing process by improving blood flow and angiogenesis in the ulcerated area, and increasing mucosal secretion [20]. NO is generated from the amino acid L-arginine by three distinct isoforms of nitric oxide synthase, namely neuronal, endothelial, and inducible [21]. The administration of L-NAME—a non-specific inhibitor of nitric oxide synthase—did not affect the gastroprotective effect of the mixture of compounds, as illustrated in Figure 4B, indicating that NO is not involved in its mechanism of action. Meanwhile, a different result was obtained with carbenoxolone, where L-NAME reversed its effect. This indicates that NO is involved in its mechanism of action, which is consistent with what has been reported in the literature [14].

It has been evidenced that ethanol’s effect on the gastric mucosa correlates with a significant decrease in the levels of non-protein sulfhydryl groups, such as glutathione (GSH) [22]. The administration of NEM—a blocker of non-protein sulfhydryl groups—reversed the gastroprotective effect of the mixture of compounds (Figure 4C), suggesting that its mechanism of action is closely related to antioxidant pathways. The decreased concentration of sulfhydryl groups could be related to glutathione oxidation following the production of toxic metabolites from ethanol, or to the binding of glutathione to the acetaldehyde produced through the oxidation of the necrotizing agent via gastric alcohol dehydrogenase activity [23]. The fact that NEM administration reversed the effect of the mixture of compounds potentially suggests that the oxidation of GSH is being prevented; either through the elimination of toxic metabolites generated by ethanol or the formation of acetaldehyde. This would block the connection with GSH, thus inhibiting lipid peroxidation. This hypothesis requires further corroboration.

Several studies have substantiated the significance of oxidative stress in the pathogenesis of gastric injury induced by ethanol. Ethanol cytotoxicity recruits inflammatory cells such as neutrophils and macrophages. An influx of reactive oxygen species (ROS) is generated by activated neutrophils and macrophages, resulting in the production of O_2_˙^−^, H_2_O_2_, HO˙, and ONOO˙. Ethanol also disrupts the microcirculation of the gastric mucosa, leading to hypoxia and further generating ROS. In this study, we found that ethanol does not induce oxidative stress, as reported by Yuan et al. [24]. This may be due to the exposure time as, after ethanol had been administered, tissue analysis was performed at 2 h. This was sufficient to observe alterations in the gastric mucosa (measured by the ulcer index), without reaching the activation of antioxidant enzymes (SOD, CAT, GPx), but with an increase in non-protein sulfhydryl groups (inhibited by NEM) [25].

## 4. Materials and Methods

### 4.1. Animals Studies

Gastroprotective activity experiments were performed in male CD1 mice with an average weight of 25–30 g and aged 7 weeks old. The antisecretory and antioxidant activity analyses were carried out in male Wistar rats with a weight between 180–220 g and aged 8 weeks old, obtained from the Unidad de Producción y Experimentación de Animales de Laboratorio (UPEAL), of Universidad Autónoma Metropolitana Campus Xochimilco, in Mexico City, Mexico. The animals were housed in polycarbonate boxes using sterilized sawdust as bedding, under controlled conditions, with a temperature of 22 ± 25 °C, airflow, a 12 h light/dark cycle, and free access to water and food (LabDiet^®^ 5012 for rats and 5001 for mice). They were fasted for 18 to 24 h before each evaluation in individual stainless-steel cages with a wire mesh floor to prevent coprophagy, but with free access to water [11]. Seven animals were used per group. All procedures involving animals were executed in accordance with the Official Mexican Standard NOM-062-ZOO-1999 [26], the technical specifications for the production, care, and utilization of laboratory animals, and the international standards for the care and utilization of laboratory animals. This study was approved by the Ethics Committee in Research of the FES Zaragoza, with the registration number FESZ/CEI/2/0/7/01/24.

### 4.2. Drugs and Compounds

The reference drugs for this study were carbenoxolone, ascorbic acid, and omeprazole, all of which were dissolved in water and administered orally. The extract and the mixture of compounds were suspended in tween 80 (0.05%) and administered via the intragastric route. L-NAME was dissolved in saline and administered intraperitoneally. NEM was dissolved in saline and indomethacin was dissolved in saline containing 5 mM NaHCO_3_; both were administered subcutaneously. All drugs were purchased from Sigma-Aldrich Co. (St. Louis, MO, USA).

### 4.3. Plant Material

During July 2023, *H. indicum* leaves were collected in Cintalapa de Figueroa, Chiapas, Mexico, which were dried at room temperature (22 ± 2 °C) in the shade. The plant was identified and registered by Manuel de Jesús Gutiérrez Morales from the Flora Department of the Chip Herbarium, which is part of the Botanical Garden of the Secretary of Environmental Protection, Housing, and Natural History of the State of Chiapas, Mexico. A specimen of the original collection can be found with the voucher number 27855.

### 4.4. Obtaining of the Extract of Hexane

A total of 7.6 kg of dried and ground *H. indicum* leaves was extracted with hexane (45 L) three times over three days at room temperature. The filtrate from each operation was concentrated in a rotary evaporator [19], yielding 200.35 g of hexanic extract. This extract was evaluated in the model of ethanol-induced gastric lesions in mice.

### 4.5. Experimental Model (Ethanol-Induced Gastric Lesions)

For this purpose, vehicle (0.05% tween 80), hexanic extract, and carbenoxolone were administered orally (0.1 mL/10 g) at different doses. Thirty minutes later, 0.2 mL of ethanol was administered orally, without regard to weight, in order to induce gastric damage. Two hours later, the animals were sacrificed in a CO_2_ chamber. Their stomachs were immediately dissected and filled with 2% formaldehyde and subsequently placed in a container with the same solution for 5 min. Finally, the stomachs were opened along the greater curvature, and the damaged area was measured with a stereoscopic microscope (×10) equipped with an ocular micrometer [14,27]. The ulcer index (mm^2^) was determined by summing up the areas of all the lesions in each animal. The percentage of gastroprotection was determined using the following formula:

$$\%\;Gastroprotection=\frac{UIC-UIT}{UIC}\times 100,$$

where UIC is the average ulcer index of the control groups and UIT is the ulcer index of each treated animal.

When the evaluation was carried out in rats, the same method was followed, with the only difference being the volume of ethanol used; in this case, it was 1 mL regardless of weight [14].

### 4.6. Fractionation of Hexanic Extract

The hexanic extract was subjected to silica gel column chromatography with large changes in polarity using hexane (100%) and hexane/ethyl acetate mixtures (9:1, 8:2, 7:3, and 5:5, respectively). In this way, five fractions were obtained (F1–F5). Biological evaluation of the fractions showed that F3 (7:3, hexane/ethyl acetate) was one of the most active and, so, was subjected to column chromatography with small changes in polarity. From fractions 102 to 133, 5.61 g of a mixture of compounds was obtained, which was responsible for the biological activity of this fraction. The crystals were characterized by mass spectrometry (MS).

### 4.7. Mass Spectrometry Analysis

Mass spectrometry analysis was conducted using DIESI-MS on the Bruker MicrOTOF-QII system with an electrospray ionization (ESI) interface (Bruker Daltonics, Billerica, MA, USA) operating in the positive and negative ion mode. An aliquot (10 µL) of the extract was re-suspended in 1 mL of methanol, filtered through a 0.25 µm polytetrafluoroethylene (PTFE) filter, and then diluted 1:100 with methanol to avoid saturation of the capillary and cone soiling. A constant volumetric flow rate (8 µL/min) was achieved using a 74900-00-05 Cole Palmer syringe pump (Billerica, MA, USA). The capillary voltage was set to 4500 V, and nitrogen was utilized as a drying and nebulizing gas at a flow rate of 4 L/min (0.4 Bar), with a gas temperature of 180 °C. Continuous spectra were collected within the *m*/*z* range of 50–3000, with a total run duration of 1 min, a scan time of 10 s, and an interscan time of 0.1 s, resulting in 6 spectra per sample.

The mass spectrometer was operated at a resolution of 11,000 (FWHM) at mass 1622.0290 *m*/*z* in positive ion modes at a capillary voltage of 4500 V (positive) and 2700 V (negative). The spectrometer underwent calibration using an ESI-TOF tuning mix calibrant from Sigma-Aldrich (Toluca, Estado de México, Mexico). Finally, precursor ion scans (MS/MS) were performed using negative and positive electrospray ionization (ESI- and ESI^+^) with an appropriate mass set. According to the obtained pattern, suitable fragments were analyzed using Bruker Compass Data Analysis 4.0 (Bruker Daltonics), which provided a list of possible elemental formulas using Generate Molecular Formula Editor, along with a sophisticated comparison of the theoretical and measured isotope patterns (σ value) for increased confidence in the suggested molecular formula (Bruker Daltonics Technical Note 008, 2004). The accuracy threshold for the confirmation of elemental compositions was established at 5 ppm.

### 4.8. Evaluation of the Possible Mechanism of Action of the Mixture of Compounds (Prostaglandins, Nitric Oxide, and Non-Protein Sulfhydryl)

In order to determine the possible involvement of prostaglandins in the mechanism of action of the mixture of compounds, the following experiments were carried out. A control group was administered subcutaneously with a saline solution containing 5 mM NaHCO_3_, and three other groups were treated with 10 mg/kg indomethacin. After 75 min, 0.05% tween 80 (0.1 mL/10 g) was given orally to the control group. Indomethacin pre-treated groups received one of three subsequent treatments independently, with either 0.05% tween 80, compound mixture (100 mg/kg), or carbenoxolone (100 mg/kg). Then, 30 min later, 0.2 mL of ethanol was administered to cause stomach damage and, 2 h later, the animals were sacrificed following the same methodology previously described, thereby determining the ulcer index [19].

For the purpose of evaluating the role of nitric oxide in the mechanism of action of the mixture of compounds, three groups of animals were pre-treated with L-NAME (70 mg/kg, 0.1 mL/10 g) intraperitoneally, while the control group was only administered a saline solution through the same route and volume. After 30 min, the pre-treated groups were administered orally (0.1 mL/10 g) one of the three treatments: 0.05% tween 80, a mixture of components (100 mg/kg), or carbenoxolone (100 mg/kg). The control group received 0.05% tween 80. Thirty minutes later, all animals were administered ethanol. Two hours later, the animals were sacrificed such that the stomachs could be removed and the ulcer index determined [19].

In order to determine whether non-protein sulfhydryl groups were part of the mechanism of action of the mixture of compounds, the control group was administered a saline solution (0.1 mL/10 g) subcutaneously, while the three other groups were pre-treated with NEM (10 mg/kg) by the same route and volume. Thirty minutes later, the control group received tween 80 at 0.05%, whereas the treated groups received a mixture of compounds or carbenoxolone. Ethanol was administered 30 min later and, 2 h later, the animals were sacrificed to determine the ulcer index [19].

### 4.9. Stomach Extraction and Preparation for Antioxidant Activity

After ethanol caused gastric lesions in the rat (1 mL), all animals were killed in a CO_2_ chamber. The stomachs were immediately removed and washed with PBS buffer (pH 7.4) in order to remove cellular debris or clots in the tissue. Briefly, for antioxidant activity, the body of each stomach was scraped and homogenized 20 times for periods of 1 min using 5 mL of a homogenizing solution (containing 250 mM sucrose, 2 mM MgCl_2_, 1 mM EGTA, and 2 mM Hepes, pH 7.4 with protease inhibitor cocktail at 0.1%) at 6000 rpm, using a tissue homogenizer and keeping the tissue temperature constant in an ice bath [28]. The homogenate obtained was centrifuged at 6000 rpm for 30 min at 4 °C, and the supernatant was divided into aliquots that were frozen at −80 °C until the antioxidant activity determinations.

The supernatant was used to quantify the content of thiobarbituric acid reactive substances (TBARS) using an assay kit (Cat #.10009055, Cayman Chemical Co., Ann Arbor, MI, USA) according to the manufacturer’s instructions. After the reaction was completed, the absorbance was detected at 532 nm [29] and the results obtained were expressed in mmol/min/mg protein. SOD was determined according to the assay kit (Cat #.706002, Cayman Chemical Co.), and the results were expressed as units (U) of SOD/mg protein. The decomposition of hydrogen peroxide (H_2_O_2_) in the presence of the enzyme catalase was estimated according to the assay kit (Cat #.707002, Cayman Chemical Co.), and the results obtained were expressed in mmol/min/mg protein. The GPx activity was determined according to the manufacturer’s specifications (Cat #.703102, Cayman Chemical Co.), and the results were expressed in nmol/min/mg of protein. The absorbance of all solutions was measured spectrophotometrically at different wavelengths corresponding to each test, according to the manufacturer’s specifications [30]. All quantifications were adjusted to mg protein in each determination.

### 4.10. Evaluation of Antisecretory Effect (Pylorus Ligation)

To assess gastric secretion, animals were fasted for 18 h and then anesthetized with a mixture of ketamine (2 mg/mL) and xylazine (5 mg/mL), administered intraperitoneally (0.1 mL/100 g). The pylorus was then surgically ligated, and animals were administered either tween 80 (0.05%), the mixture of compounds (100 mg/kg), or omeprazole (30 mg/kg) orally. They were sacrificed 4 h later to dissect the stomachs and collect gastric contents, which were centrifuged at 3000 rpm for 5 min to determine the volume and pH (with a pH meter) of the supernatant [31].

## 5. Conclusions

In summary, the present study identified two groups of compounds that are responsible for the gastroprotective activity of the hexanic extract of *Heliotropium indicum*. The first refers to 1-acyl-glycerol components, while the second refers to retinyl β-glucuronide derivatives. It was further determined that the mechanism of action of the extract involves non-protein sulfhydryl groups.

## Figures and Tables

**Table 1:** Fractionation of compounds.

MW_obs_	MW_exact_	Formula	Error (ppm)	MSigma	%RA
347.2532	347.2557	C_20_H_36_O_3_ [M+Na]^+^	−7.2	17.5	10.8
361.2684	361.2713	C_21_H_38_O_3_ [M+Na]^+^	−8.1	10.2	5.1
375.2783	375.2870	C_22_H_40_O_3_ [M+Na]^+^	11.1	8.9	2.3
389.2802	389.3026	C_23_H_42_O_3_ [M+Na]^+^	3.3	20.5	1.4
403.2451	403.2479	C_24_H_35_O_5_ [M+H]^+^	−6.9	4.6	2.4
417.2608	417.2636	C_25_H_37_O_5_ [M+H]^+^	6.5	11.6	7.2
431.2752	431.2792	C_26_H_39_O_5_ [M+H]^+^	9.2	15.4	10.9
463.2642	463.2666	C_26_H_39_O_7_ [M+H]^+^	5.2	4.7	20.3
477.2794	477.2823	C_27_H_41_O_7_ [M+H]^+^	11.1	10.7	16.3
491.2947	491.3003	C_28_H_43_O_7_ [M+H]^+^	−11.4	9.0	23.2

**Table 2:** Antisecretory activity.

Treatment	Dose (mg/kg)	n	Volume mL	pH
Control	---	7	1.68 ± 0.51	1.89 ± 0.32
Mixture of compounds	100	7	1.02 ± 0.03 *	2.3 ± 0.41
Omeprazole	30	7	1.57 ± 0.10	5.98 ± 0.24 ^#^

Data are expressed as the mean ± SEM. * *p* < 0.05, based on the Kruskal−Wallis test followed by Dunn’s multiple comparison. ^#^ *p* < 0.05 vs. control, ANOVA followed by Holm–Sidak multiple comparison versus control.

## Data Availability

The datasets analyzed during the current study are available from the corresponding author upon reasonable request. The data are not publicly available due to restrictions.
